# Malaria parasite carriage before and two years after the implementation of seasonal malaria chemoprevention: a case study of the Saraya health district, southern Senegal

**DOI:** 10.12688/wellcomeopenres.17888.1

**Published:** 2022-07-05

**Authors:** Isaac Akhenaton Manga, Mamadou Sarifou BA, Fassiatou Tairou, Amadou Seck, Ekoue Kouevidjin, Doudou Sow, Khadime Sylla, Magatte Ndiaye, Fatou Ba Fall, Alioune Babara Gueye, Ibrahima Diallo, Médoune Ndiop, Mady Ba, Roger Clément Tine, Omar Gaye, Babacar Faye, Jean Louis Abdourahim Ndiaye

**Affiliations:** 1 Parasitology-Mycology Department, Faculty of Medicine, Pharmacy and Ondontology, Cheikh Anta Diop University of Dakar, Dakar, Senegal; 2 Ministry of Health and Social Action, Dakar, Senegal; 3 National Malaria Control Program, Dakar, Senegal; 4 Service of Parasitology Mycology, Department of Medical Biology, UFR Santé/University Iba Der Thiam, Thies, Senegal

**Keywords:** seasonal malaria chemoprevention, Plasmodium, carriage, Senegal, malaria

## Abstract

**Background** **:** Seasonal malaria chemoprevention (SMC) has been adopted and implemented in the southern regions of Senegal in children aged between three and 120 months since 2013. Scaling up this strategy requires its evaluation to assess the impact. This study was carried out to determine the dynamics of *Plasmodium falciparum* carriage before and after two years of SMC implementation.

**Methods** **:** Four household surveys were conducted in villages in the health district of Saraya, which is a SMC implementation area in Senegal. These villages were selected using probability proportional to size sampling. Each selected village was divided into segments containing at least 50 children. In each segment, a household questionnaire was administered to the parents or legal representatives of children aged three to 120 months. Blood smears were collected to determine *P*. *falciparum* prevalence by microscopy one month before the first round of SMC, one month after the last round of the first SMC campaign and two years after the start of the implementation.

**Results** **: **A total of 2008 children were included with a mean average age of 4.81 (+/-2.73) years. Of the study population, 50.33% were more than five years old and 50.3% were male. In 2013, mosquito net ownership was 99.4 % before the SMC campaign and 97.4% after. In 2015, it was 36.6% before and 45.8% after the campaign. In 2013, the prevalence of plasmodium carriage was 11.8% before and 6.1% after the SMC campaign. In 2015, the prevalence was 4.9% before the administration of SMC and this increased up to 15.3% after. Malaria prevalence was high among children over five years old and in boys.

**Conclusions** **:** The decrease in *Plasmodium falciparum* parasite prevalence, which subsequently increased after two years of SMC implementation in this study, suggests adding an extra cycle of the SMC or adjusting the administration period.

## Introduction

Seasonal malaria chemoprevention (SMC) is a targeted strategy in addition to the existing malaria control interventions. Previously known as intermittent preventive treatment of malaria in children (IPTc), SMC involves monthly antimalarial treatment with sulfadoxine-pyrimethamine plus amodiaquine (SPAQ) for up to four months to prevent malaria. It has been recommended by the World Health Organization (WHO) since March 2012, for children aged 3–59 months living in areas with intense and highly seasonal malaria transmission in the Sahel sub-region ( WHO. Report of the Technical consultation on SMC, 2011.), ^1^. This intervention has been shown to be effective ^2^, cost-effective ^3^, safe and feasible for malaria prevention among children under five years ^4– 7^. This strategy has been adopted in many countries in sub-Saharan Africa ^1, 8^ for implementation in areas that meet the eligibility criteria ( WHO. Report of the Technical consultation on SMC, 2011.), ^9^. In order to optimize the fight against malaria, Senegal adopted SMC as one of malaria prevention strategies but extended the coverage to children aged between three and 120 months. This decision was based on the results from other studies conducted within the country that have shown that children aged 5–10 years old were as vulnerable as those under five years old ^1, 3– 5, 7, 9^. SMC was implemented in 2013 in the southeastern part of the country. To be more effective and performant, the door-to-door strategy was adopted, and drugs were administered by community volunteers. The effectiveness of this method has been demonstrated in studies conducted in many countries including Senegal, Gambia and Mali ( WHO. Report of the Technical consultation on SMC, 2011.), ^2, 7^. WHO also advocated its regular surveillance based on epidemiological and biological arguments ^9^. In this context, this study was conducted to assess the dynamics of *Plasmodium* carriage at the beginning and two years after the implementation of SMC in southern Senegal. The objective was to determine the prevalence of malaria before and after the first two mass distribution campaigns of SMC in order to assess the impact of this strategy.

## Methods

### Study area

**Figure 1.**
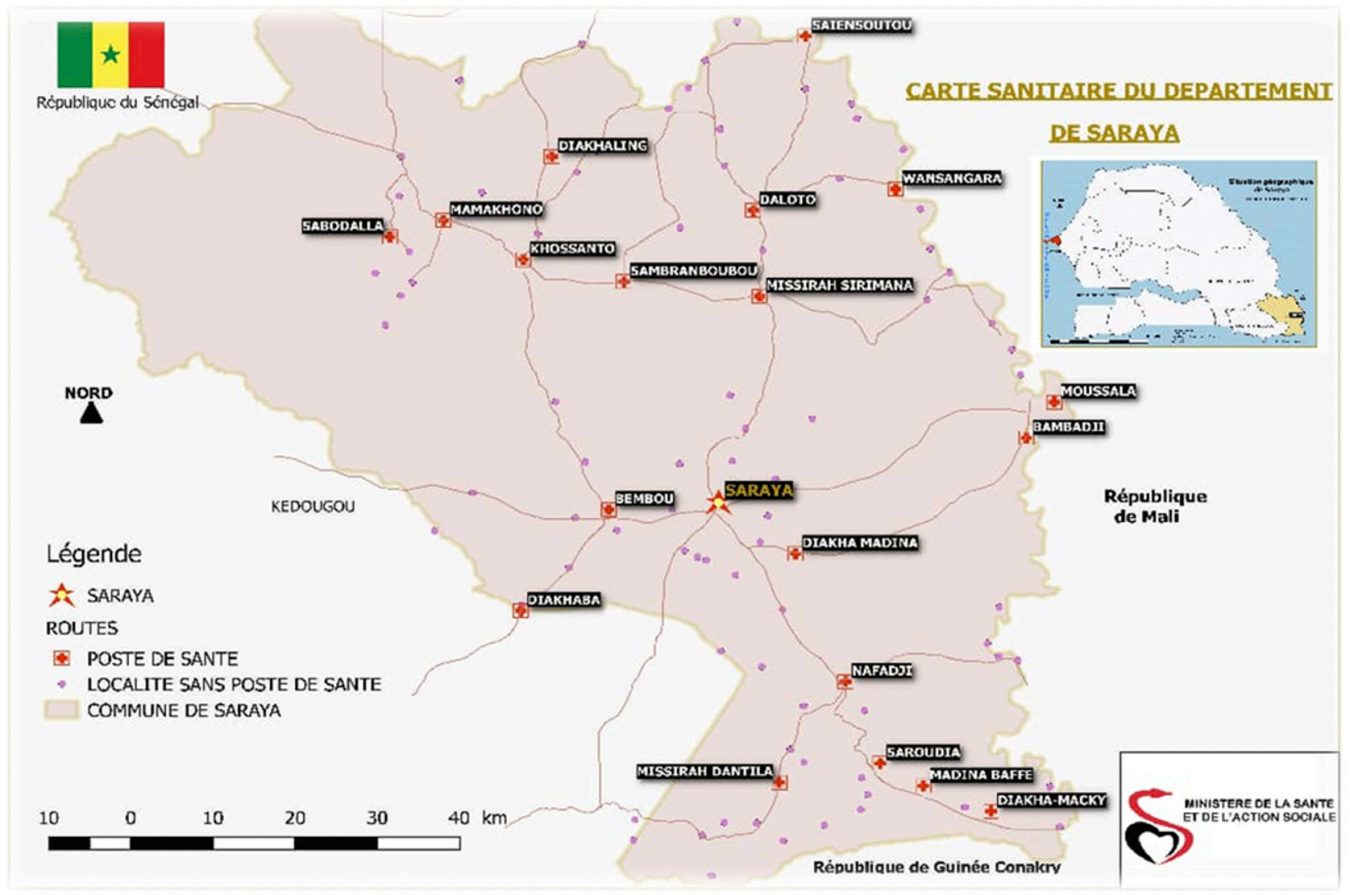
Map of the health district of Saraya in the Kédougou region of Senegal. (The map was produced by the Saraya health district which also allowed us to include it in this article). 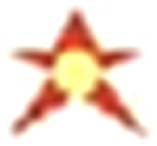
Saraya, 
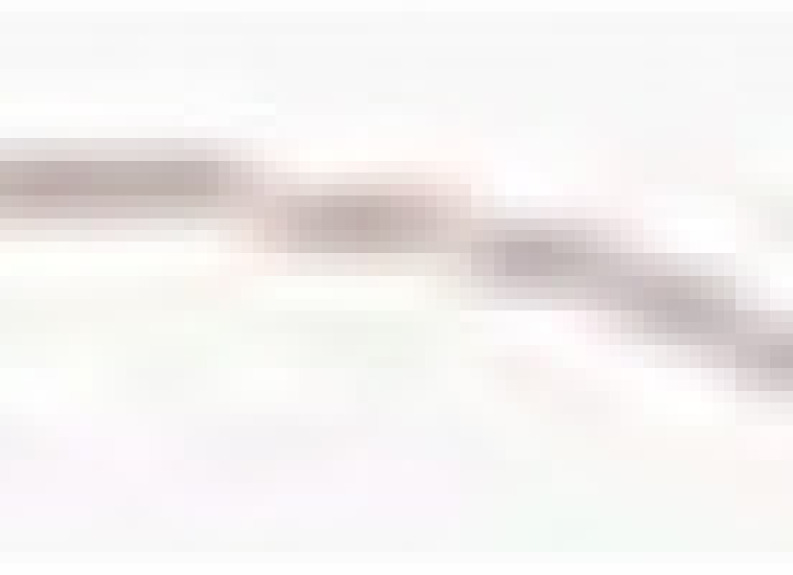
Road, 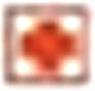
Health Post, 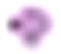
Locality without Health Post, 
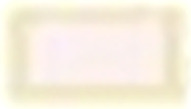
Commune of Saraya

Four household surveys were performed targeting children aged three to 120 months living in the health district of Saraya. Saraya district is located in Kedougou region, south-east Senegal, which covers an area of 6837 km ^2^_._ It is bordered in the south by the Republic of Guinea and in the east by the Republic of Mali ( Figure 1: Source: Saraya Health District) ^10^.

Malaria is a major public health problem because the overall incidence of malaria in the Kédougou region was 36.9% in 2019, with a proportional morbidity rate of 27% and the same proportion for the mortality rate. The latter was higher in children under five years of age (50%). Malaria is also endemic with a seasonal upsurge in transmission between July and November ( NMCP. Epidemiological report, 2019.). Therefore, Saraya district meets the eligibility criteria for the implementation of SMC as recommended by WHO ^9^.

### Study design and population

Four cross sectional household surveys were conducted during this study. In 2013, baseline surveys were carried out in August while SMC mass campaigns were performed in November and December. The surveys were conducted again one month after the campaign in January 2014. Two other surveys were conducted in August and December 2015 while SMC campaigns were performed from September to November i.e., one month before and one month after the last cycle of SMC. The study population consisted of three to 120 months old children living in Saraya district.

The study inclusion criteria were the parents' consent, apparent good health, absence of fever in the week prior to our visit, being between three and 120 months of age and residing in the study area for the entire period of SMC administration.

Given the malaria prevalence of about 15%, we assumed that 50 children in 10 villages, and with a non-response rate of 10%, the sample size was estimated to 550 children per survey. These children were recruited in villages where selection was based on probability proportional to size of the population. Each selected village was divided into different segments using the formula S = 1 + int (0.38N / 50) where "S" is the number of segments, "int" is the integral, "N" is the total population of the village, "0.38" is the factor to be multiplied to the general population in order to get the total number of eligible children for our study, and "50" is the desired number of eligible children per segment.

The villages were divided in segments, using the village maps provided by the National Agency for Statistics and Demography (NASD) and the Geography Department of University Cheikh Anta Diop de Dakar (UCAD). Based on the map and the approximate population, the village was divided into the number of segments calculated, in line with the natural boundaries (such as roads and paths). One of the segments was then drawn at random from the center of the village for the household survey and all the households in that segment were visited. If in this segment, the required number was not reached, the field team moved on to the next neighbor on the list.

### Data collection

A standardized questionnaire was administered to parents or care givers/guardians of each selected child to collect socio-demographic data and to assess the use of malaria prevention measures like the use of mosquito bed nets ^11^. A blood smear was taken to determine the prevalence of malaria using microscopy.

### Laboratory methods

***Parasitological assessment.*** For each child, finger-prick blood samples were collected for thick and thin smear tests to determine malaria prevalence using microscopy. The staining and determination of the parasite density when a slide was positive was done according to the recommendations of the NMCP in its guide for the biological diagnosis of malaria (NMCP. National biological diagnosis guide for malaria in Senegal, 2018). The reading was done by technicians from two different laboratories (Parasitology-mycology department of the Cheikh Anta Diop University of Dakar and that of the Fann University Hospital Centre, both in Senegal) according to the recommendations of the national guideline for the biological diagnosis of malaria ( NMCP. National diagnostic guidelines for malaria, 2018.). A comparison of the results of the two readings was made before selecting one. When they were identical, the given one was retained. On the other hand, when they were discordant (opposite results or a difference in parasite density greater than 25% for a positive result), a third reading was used. The final result was the one having two readings with the same result.

### Statistical methods

The data collected were entered into a Microsoft Excel 2019, 16.60 (22041000) **(**RRID:SCR_016137) file and analyzed using Epi Info 7.1.3.3 software **(**RRID:SCR_021682). Quantitative variables are expressed in terms of the mean with their standard deviation. Qualitative variables are expressed as percentages with their confidence intervals. The different variables were analyzed according to the period of the surveys, age groups and gender. The significance threshold for comparisons between the different groups was set at 0.05. The SMC coverages considered in this study were those of the latest rounds given by the NMCP after the 2013 ( NMCP. Epidemiological report, 2014.) and 2015 ( NMCP. Epidemiological report, 2015.) administration campaigns. 

### Ethical considerations

This study was approved by the National Health Research Ethics Committee (Approval number CNERS SEN13/57). Written informed consent was obtained from the parents or legal representatives of potential child participant prior to enrolment in the study. To respect confidentiality, an identification code was given to each participant.

## Results

### Characteristics of the study population

Overall, 2008 children aged three to 120 months were enrolled in this study. This was made up of 498 and 491 children before and after the SMC campaign in 2013, while 547 and 472 children were enrolled before and after the 2015 campaign. Children under five years old represented 52.8% [CI 95%: 48.3–57.3%] of the children in the first survey, 43.6% [CI 95%: 38.2–48.1%] in the second, 47.3% [CI 95%: 43.1–51.6%] in the third and 51.5% [CI 95%: 46.9–56.1%] in the 2015 post-campaign survey. Young girls were predominant during the pre-campaign of 2013, 50.8% [CI 95%: 46.3–55.3%] and the two 2015 surveys, with 51.2% [CI 95%: 46.9–55.5%] for the pre-campaign and 51.91% [CI 95%: 47.3–56.5%] for the post-campaign. SMC coverage after the last round was 96.7% after the 2013 mass administration campaign and 90.4% after the 2015 campaign. Children who slept under a bed net the week before the surveys were higher in 2013: 99.4% [CI 95%: 98.1–99.8%] and 97.3% [CI 95%: 95.4–98.5%] respectively for the pre- and post-campaign compared to 2015, 36.6% [CI 95%: 32.6–40.8%] and 45.8% [CI 95%: 41.2–50.4%] ( Table 1) ^10^.

**Table 1.** Distribution of the study population by survey period, age, gender and mosquito net use.

	Pre-campaign 2013 (N=498)	Post-campaign 2013 (N=491)	Pre-campaign 2015 (N=547)	Post-campaign 2015 (N=472)
Age group				
**Less than 5 years**	263 (52.8% ; CI 95%=48.3–57.3%)	214 (43.6% ; CI 95%=38.2–48.1%)	259 (47.3% ; CI 95%=43.1–51.6%)	243 (51.5% ; CI 95%=46.9–56.1%)
**5–-10 years**	235 (47.2% ; CI 95%=42.7–51.7%)	277 (56.4% ; CI 95%=51.9–60.8%)	288 (52.7% ; CI 95%=48.4–56.9%)	229 (48.5% ; CI 95%=43.9–53.1%)
Gender				
**Boys**	245 (49.2% ; CI 95%=44.7–53.7%)	267 (54.4% ; CI 95%=49.8–58.8%)	267 (48.8% ; CI 95%=44.6–53.1%)	227 (48.1% ; CI 95%=43.5–52.7%)
**Girls**	253 (50.8% ; CI 95%=46.3–55.3%)	224 (45.6% ; CI 95%=41.2–50.1%)	280 (51.2% ; CI 95%=46.9–55.5%)	245 (51.9% ; CI 95%=47.3–56.5%)
Prevention				
**Slept under a** **mosquito net week** **before**	495 (99.4% ; CI 95%=98.1–99.8%)	478 (97.3% ; CI 95%=95.4–98.5%)	200 (36.6% ; CI 95%=32.6–40.8%)	216 (45.8% ; CI 95%=41.2–50.4%)

### Parasite carriage

Microscopic examination of the blood smear during the different household surveys showed a *Plasmodium* carriage rate of 11.8% in the first survey, 6.1% in the second, 4.9% in the third and 15.2% in the last. *Plasmodium* carriage was higher in older children (5–10 years) in all surveys. The prevalence was 7.2% in the 2013 pre-campaign, 4.7% in the 2013 post-campaign, 3.3% in the 2015 pre-campaign, and 9.1% in the 2015 post-campaign survey in older children. Among children under five years old, the prevalences were 4.6%; 1.4%; 1.6%; and 6.1% for the 2013 pre- and post-campaigns and 2015 pre- and post-campaigns, respectively. This difference in proportion between the two age groups was statistically significant for the different surveys (p = 0.01 for the pre- and post-campaign of 2013, and p = 0.02 for the 2015 post campaign) except for the 2015 pre-campaign survey where p = 0.07.

According to gender, *Plasmodium* carriage in girls was 6% and 2.6% respectively for the pre- and post-campaigns of 2013 and, 2.9% and 8.2% respectively for those of 2015. Among young boys, it was 5.8% before the campaign in 2013, and 3.5% after; 2% and 7% respectively before and after the 2015 campaign. Despite these differences, no statistical significative association was found between parasite carriage and gender in the surveys (p = 0.497; p = 0.473; p = 0,199; p = 0.34 respectively for the pre- and post-campaign in 2013 and 2015).

In the 2013 pre-campaign survey, all children with a positive slide (59 i.e., a prevalence of 11.8%) had slept under a mosquito net the night before the survey. The survey conducted one month after the administration of SMC in 2013 found that of the 30 positive subjects only two (6.7%) had not used net the day before the survey. In 2015, the survey conducted one month before the SMC campaign showed that of the 27 participants that had *Plasmodium*-positive slides, 16 (59.2%) had not slept under a net the night before the survey. The survey conducted one month after the 2015 campaign showed that 29 (40.3%) participants of the 72 with positive slides, had not slept under a bed net the previous night. *Plasmodium falciparum* was the prevalent species in this study regardless of the survey. The presence of other species such as *Plasmodium malariae* and *Plasmodium ovale* was also noted at low prevalence in the different surveys. Mixed infections with *P. falciparum* and *P. malariae* were found during the pre- and post-campaign surveys in 2015. *P. falciparum* gametocytes were identified on positive slides in the survey conducted in 2013 before SMC (0.6%), but also in the two surveys in 2015 (0.2% and 1.9% respectively before and after the campaigns) ( Table 2) ^10^.

**Table 2.** Distribution of *Plasmodium* carriage by survey period, age, sex, possession and use of a mosquito net, plasmodial species and presence of gametocytes.

	Pre-campaign 2013 (N=498)	Post-campaign 2013 (N=491)	Pre-campaign 2015 (N=547)	Post-campaign 2013 (N=472)
**Plasmodium parasite** **carriage**	11.8% (59)	6.1% (30)	4.9% (27)	15.2% (72)
Age group				
** • Less than 5 years**	4.6% (23)	1.4% (7)	1.6% (9)	6.1% (29)
** • 5–10 years**	7.2% (36)	4.7% (23)	3.3% (18)	9.1% (43)
• p-value *	**0.01**	**0.01**	0.07	**0.02**
Sex				
** • Girls**	6% (30)	2.6% (13)	2.9% (16)	8.3% (39)
** • Boys**	5.8% (29)	3.5% (17)	2% (11)	7% (33)
• p-value *	0.497	0.473	0.199	0.34
Slept under LLIN the night before the survey				
** • No**	0	0.4% (2)	2.9% (16)	6.1% (29)
** • Yes**	11.8% (59)	5.7% (28)	2% (11)	9.1% (43)
• p-value *	0.301	0.127	0.38	0.004
Parasite species				
*** • P. falciparum***	11.5% (57)	6.1% (30)	4% (22)	14.8% (70)
*** • P. falciparum* and** ***P. malariae***	-	-	0.4% (2)	0.2% (1)
*** • P. malariae***	0.4% (2)	-	-	-
*** • P. ovale***	-	-	0.5% (3)	-
**Gametocytes**	0.6% (3)	-	0.2% (1)	1.9% (9)

*p for comparison between different variables for the same survey

## Discussion

This study determined the variations in *Plasmodium* carriage during the first two years of the scaling up of the SMC in a mass campaign in Senegal. It was conducted in the region of Kedougou where malaria is a real problem for public health. A variation in the number of children recruited during the four surveys was noted during this study.

A decrease in parasite carriage was observed before the start of the SMC in 2013 and the survey one month after the mass administration campaign the same year, but also during the pre-campaign survey of 2015. This decrease could be explained by the higher SMC coverage rates in 2013 (95%) and 2014 (98%) ( NMCP. Annual report of NMCP activities 2014 in Senegal, 2014.). It could also be explained by the free mass distribution of LLINs organized by the NMCP throughout the country between 2012 and 2013 ( NMCP. Report of the national mass distribution campaign of LLINs in Senegal, 2014.). The decrease in the parasite carriage observed in the first three surveys also reflects the decrease in malaria-related morbidity and mortality noted between 2013 and 2014 ( NMCP. Epidemiological report, 2014.). This finding was shown in a study conducted in Senegal in 2009. This study estimated a considerable decrease in prevalence over the years of SPAQ administration ^7^.

The rebound in parasitemia observed during the household survey one month after the 2015 campaign, despite good SMC coverage, could be explained by an increase in rainfall compared to 2014 ( NMCP. Epidemiological report, 2015.). It could also be explained by the gradual decline in net usage rates noted between 2013 and 2015 in this study. This increase was also felt by an increase of malaria morbidity and mortality in the 2015 NMCP report ( NMCP. Epidemiological report, 2015.).

The higher parasite carriage in children over five years of age found in our study confirms the need to broaden the SMC target in Senegal as demonstrated by previous studies conducted in the country prior to the adoption of the strategy (WHO. SMC background documents, 2012. https://www.who.int/malaria/mpac/feb2012/smc_bibliography.pdf), ^7, 12^. In Niger, a study assessing the dynamics of *Plasmodium* carriage after the implementation of SMC also found that children over five years of age were those with the highest prevalence. This study reported higher parasite prevalence than those found in our study ^13^. *P. falciparum* was the most frequent species found in the present study and this further supports the choice of this area for the implementation of the SMC as recommended by WHO in its field guide for implementation ^9^. As in this study, other species of *Plasmodium* parasites such as *P. malariae* and *P. ovale* were also found in other studies conducted in the Kedougou region where our study site was located ^14, 15^. The presence of gametocytes in children, even if it is more important to ascertain their presence in children under five years old, during the different surveys, proves that they constitute a reservoir of parasites. The addition of low dose primaquine to SMC is an option to be considered. The effect of this medication on gametocytes has been proven by numerous studies, including one from Senegal ^16^ .

## Conclusions

An evaluation of the parasite carriage of *Plasmodium* during the first two years of the implementation of SMC in Senegal showed that this strategy led to a reduction in parasitemia. However, the increase in parasitemia during the last survey, probably linked to a longer rainy season, implies an adjustment of the administration period or the addition of another administration cycle. The presence of gametocytes noted in this study despite the administration of SMC implies the need of optimizing the strategy by combining SMC with transmission blocking tools such as primaquine, ivermectin, a new generation of mosquito nets or adding seasonal vaccination of the recently approved RTS,S malaria vaccine for best impact.

## List of abbreviations

SMC       seasonal malaria chemoprevention 

WHO     World Heath Organization

NASD     National Agency for Statistics and Demography

HCP        Home care providers

NMCP     National Malaria Control Program

LLIN       Long-lasting impregnated net

UCAD     Université Cheikh Anta Diop de Dakar

IPTc         intermittent preventive treatment of malaria in children

SPAQ       sulfadoxine-pyriméthamine plus amodiaquine

## Data availability

### Underlying data

Dryad: Underlying data for ‘Malaria parasite carriage before and two years after the implementation of seasonal malaria chemoprevention: a case study of the Saraya health district, southern Senegal’ https://doi.org/10.5061/dryad.6m905qg21 ^11^

This project contains the following underlying data:

- Data file: Saraya plasmodium carriage 2013–2015.xlsx
- Supplementary file: Readme.Saraya.2013et 2015.docx

Data are available under the terms of the Creative Commons Zero “No rights reserved” data waiver (CC0 1.0 Public domain dedication).

Zenodo: Underlying data for ‘Malaria parasite carriage before and two years after the implementation of seasonal malaria chemoprevention: a case study of the Saraya health district, southern Senegal’ https://doi.org/10.5281/zenodo.6634394 ^10^

This project contains the following underlying data:

- Figure 1: Map of the health district of Saraya in the Kédougou region of Senegal. (The map was produced by the Saraya health district which also allowed us to include it in this article)
- Table 1: Distribution of the study population by survey period, age, gender and mosquito net use
- Table 2: Distribution of *Plasmodium* carriage by survey period, age, sex, possession and use of a mosquito net, plasmodial species and presence of gametocytes

Data are available under the terms of the Creative Commons Attribution 4.0 International license (CC-BY 4.0)

### Extended data

Dryad: Extended data for ‘Malaria parasite carriage before and two years after the implementation of seasonal malaria chemoprevention: a case study of the Saraya health district, southern Senegal’ https://doi.org/10.5061/dryad.6m905qg21 ^11^.

This project contains the following extended data:

- Questionnaire: children 3–120 months.pdf

Data are available under the terms of the Creative Commons Zero “No rights reserved” data waiver (CC0 1.0 Public domain dedication).

## Consent

Written informed consent for publication of the participants details was obtained from the parents or guardian of the participant.
