# Supplementary material for: Malaria parasite carriage before and two years after the implementation of seasonal malaria chemoprevention: a case study of the Saraya health district, southern Senegal
Source: Wellcome Open Res. 2022 Oct 11;7:179. Originally published 2022 Jul 5. [Version 2] doi: 10.12688/wellcomeopenres.17888.2 (PMC10375055; doi:10.12688/wellcomeopenres.17888.2)
Supplement: Peer review history

# Peer review: Reviewer response for version 2

**DOI:** 10.21956/wellcomeopenres.20462.r61245

**Published:** 2023-07-27

**Authors:** Benoit Malleret

**Affiliations:** 1 National University of Singapore, Singapore, Singapore

The manuscript “Malaria parasite carriage before and two years after the implementation of seasonal malaria chemoprevention: a case study of the Saraya health district, southern Senegal” by Manga *et al.* provides valuable insights into the potential of Seasonal Malaria Chemoprevention (SMC) for malaria control/eradication in Senegal. The study evaluates the prevalence of malaria parasite carriage before and after the implementation of SMC in the Saraya health district, southern Senegal. While the authors have presented a comprehensive analysis, there are a few key points that need to be addressed to further enhance the validity and impact of their findings. Overall, the study carries significant implications for malaria eradication efforts in Senegal. Addressing the points mentioned below will strengthen the methodological aspects of the research and increase the impact of the findings.

 Methodology:

 One critical aspect that requires clarification is the limit of detection for parasitemia. Given that the study aims to assess the impact of SMC on malaria parasite carriage, it is essential to specify the sensitivity threshold of the methods used for parasite detection. The introduction of ultra-sensitive PCR (uPCR) has revolutionized the detection of *Plasmodium* spp., particularly for asymptomatic malaria patients with parasitemia levels below the detection limits of microscopy.

 Minor Comments:

 Figure 1 Resolution: The authors should address the issue of low resolution in Figure 1, which hinders the proper display of the Senegal map in the insert. Enhancing the resolution will improve the clarity of the map and facilitate better understanding for readers.

Is the work clearly and accurately presented and does it cite the current literature?

Yes

If applicable, is the statistical analysis and its interpretation appropriate?

Yes

Are all the source data underlying the results available to ensure full reproducibility?

Yes

Is the study design appropriate and is the work technically sound?

Yes

Are the conclusions drawn adequately supported by the results?

Yes

Are sufficient details of methods and analysis provided to allow replication by others?

Yes

Reviewer Expertise:

NA

I confirm that I have read this submission and believe that I have an appropriate level of expertise to confirm that it is of an acceptable scientific standard.

# Peer review: Reviewer response for version 2

**DOI:** 10.21956/wellcomeopenres.20462.r52752

**Published:** 2022-11-24

**Authors:** Innocent Mbulli Ali

**Affiliations:** 1 MARCAD Program, The Biotechnology Centre, University of Yaoundé 1, Yaoundé, Cameroon; 2 Department of Biochemistry, University of Dschang, Dschang, Cameroon

Version 2 of the article appears better and and overall improves the quality of reporting done by the authors.

Is the work clearly and accurately presented and does it cite the current literature?

Partly

If applicable, is the statistical analysis and its interpretation appropriate?

Partly

Are all the source data underlying the results available to ensure full reproducibility?

Yes

Is the study design appropriate and is the work technically sound?

Yes

Are the conclusions drawn adequately supported by the results?

Yes

Are sufficient details of methods and analysis provided to allow replication by others?

Yes

Reviewer Expertise:

Malaria Molecular/Serological diagnostics, Molecular Epidemiology of Drug Resistance, Malaria Microscopy

I confirm that I have read this submission and believe that I have an appropriate level of expertise to confirm that it is of an acceptable scientific standard.

# Peer review: Reviewer response for version 1

**DOI:** 10.21956/wellcomeopenres.19819.r51961

**Published:** 2022-10-04

**Authors:** Innocent Mbulli Ali

**Affiliations:** 1 MARCAD Program, The Biotechnology Centre, University of Yaoundé 1, Yaoundé, Cameroon; 2 Department of Biochemistry, University of Dschang, Dschang, Cameroon

**Summary:**

 The research report presented by Manga IA et al presents an important finding in Senegal addressing a key aspect of the impact of seasonal malaria chemoprevention strategy in Sahel regions of Africa. These findings will hopefully contribute to the growing body of literature on the widespread implementation of SMC in Africa. A few comments to improve the quality of the work are presented below. Once the comments have been addressed, the whole article should be proofread for english language.

**Introduction and Methods:**

 The research work is clearly presented but lack some elements that improve the accuracy of background information.

 First the first paragraph of the manuscript describes SMC as a targeted strategy but does not say clearly the target of the strategy. This should be clear before the third sentence of this introduction to provide a clear context reading further.

 Was this study conducted as ancillary to another study? If yes, this should be indicated to provide more context.

- Is the study design appropriate and does the work have academic merit?

The study design is appropriate for testing parasite rates in the community in the context of seasonal malaria chemoprevention. 

- Are sufficient details of methods and analysis provided to allow replication by others?

The statistics analysis section needs to be re-written to identify what variables the authors referred to, and how they were handled. In addition, there is a need to precise what statistical tests were used to compare parasite rates and justification for the choice of statistical methods provided. The authors should proofread the manuscript/article for english language and tenses used. There needs to be consistency in language to ease reading.

 The section on ethical considerations should be detailed. Did the authors obtain assent for children 9-10 years old? If yes, how, if no, why not?  In addition, the authors are dealing with huge datasets. Did the authors obtain permission for potential publication of research results?

  

- Are all the source data underlying the results available to ensure full reproducibility?

Yes, this is an important criterion for reproducibility and integrity in scientific research. 

 In the results section table 2, the p values reflect a comparison of parasite rates between the two age groups or between the pre- and post surveys? This needs to be clearly reflected. perhaps the table title should change to clearly and specifically reflect the statistics. I will suggest that comparisons for pre- post surveys be separated from comparisons between age groups of the study population. 

**Conclusion:**

- Are the conclusions drawn adequately supported by the results?

The conclusions are adequately supported by the results obtained. Perhaps, the Authors should formulate a recommendation for a more formal assessment superposing epidemiological and rainfall data to support the proposal to add another cycle of the SP and AQ administration.

Is the work clearly and accurately presented and does it cite the current literature?

Partly

If applicable, is the statistical analysis and its interpretation appropriate?

Partly

Are all the source data underlying the results available to ensure full reproducibility?

Yes

Is the study design appropriate and is the work technically sound?

Yes

Are the conclusions drawn adequately supported by the results?

Yes

Are sufficient details of methods and analysis provided to allow replication by others?

Yes

Reviewer Expertise:

Malaria Molecular/Serological diagnostics, Molecular Epidemiology of Drug Resistance, Malaria Microscopy,

I confirm that I have read this submission and believe that I have an appropriate level of expertise to confirm that it is of an acceptable scientific standard, however I have significant reservations, as outlined above.

# Response

**Published:** 2022-10-06

**Authors:** Isaac Akhenaton MANGA

**Affiliations:** Faculté de Médecine FMPO/UCAD, Senegal

Thank you for the suggestions and recommendations to improve the quality of this article. We have taken them into account in the new version resubmitted to the journal.

# Response

**Published:** 2022-10-24

**Authors:** Isaac Akhenaton MANGA

**Affiliations:** Faculté de Médecine FMPO/UCAD, Senegal

**Introduction and method**

 We have added the WHO recommended SMC target as you suggested.

 Yes, this study was also conducted to assess the impact of this strategy on the molecular markers of Plasmodium resistance to the drugs used. We think that adding it in the context could be confusing. That is why we have not added it in the new version submitted for publication. 

**Statistical analysis**

 We have rewritten this section to clarify the nature of some of the variables. In addition we have also given the tests used for each comparison.

 The p-value given in Table 2 corresponds to a comparison of the different categories of each survey as indicated at the bottom of the table. 

**Ethical considerations**

 We only asked for parental consent for their child's participation in this study. We thought that assent should be obtained in adolescence, i.e. at an age above 10 years.

 The use of other data besides the study data was done with the permission of the competent authorities. The CMS coverage data used here was only possible with the endorsement of the NMCP, some of whose members are among the authors of this article.

**Conclusion**

 The proposal to link our data with rainfall and epidemiological data is very relevant to justify the need to adapt the administration cycles. This perspective will be taken into account in the correction of the next version.
